# Association of C-reactive protein with histological, elastographic, and sonographic indices of non-alcoholic fatty liver disease in individuals with severe obesity

**DOI:** 10.1186/s41043-023-00372-8

**Published:** 2023-04-07

**Authors:** Tannaz Jamialahmadi, Simona Bo, Mitra Abbasifard, Thozhukat Sathyapalan, Ali Jangjoo, Seyed Adel Moallem, Wael Almahmeed, Sorour Ashari, Thomas P. Johnston, Amirhossein Sahebkar

**Affiliations:** 1 Surgical Oncolgy Research Center, Mashhad University of Medical Sciences, Mashhad, Iran; 2 Applied Biomedical Research Center, Mashhad University of Medical Sciences, Mashhad, Iran; 3 Department of Medical Sciences, AOU Città della Salute e della Scienza di Torino, University of Turin, Turin, Italy; 4 Immunology of Infectious Diseases Research Center, Research Institute of Basic Medical Sciences, Rafsanjan University of Medical Sciences, Rafsanjan, Iran; 5 Department of Internal Medicine, Ali-Ibn Abi-Talib Hospital, School of Medicine, Rafsanjan University of Medical Sciences, Rafsanjan, Iran; 6 Academic Diabetes, Endocrinology and Metabolism, Hull York Medical School, University of Hull, Hull, UK; 7 Department of Pharmacology and Toxicology, College of Pharmacy, Al-Zahraa University for Women, Karbala, Iraq; 8 Department of Pharmacodynamics and Toxicology, School of Pharmacy, Mashhad University of Medical Sciences, Mashhad, Iran; 9 Heart and Vascular Institute, Cleveland Clinic Abu Dhabi, Abu Dhabi, United Arab Emirates; 10 Division of Pharmacology and Pharmaceutical Sciences, School of Pharmacy, University of Missouri-Kansas City, Kansas City, MO USA; 11 Biotechnology Research Center, Pharmaceutical Technology Institute, Mashhad University of Medical Sciences, Mashhad, Iran

**Keywords:** Fatty liver, Steatohepatitis, Liver fibrosis, Fatty liver, Biopsy, Elastography, Inflammation

## Abstract

**Background:**

Inflammation is critical in the pathogenesis of non-alcoholic fatty liver disease (NAFLD). hs-CRP, an inflammatory marker, is considered one of the prognostic predictors of hepatic damage progression in NAFLD in some studies.

**Methods:**

We assessed the concordance of hs-CRP concentrations and liver steatosis, steatohepatitis, and fibrosis based on elastography, sonography and liver biopsy findings in patients with severe obesity undergoing bariatric surgery.

**Results:**

Among 90 patients, 56.7% showed steatohepatitis and 8.9% severe fibrosis. Hs-CRP were significantly associated with liver histology in an adjusted regression model (OR 1.155, 95% CI 1.029–1.297, *p* = 0.014; OR 1.155, 1.029–1.297, *p* = 0.014; OR 1.130, 1.017–1.257, *p* = 0.024 for steatosis, steatohepatitis, and fibrosis, respectively). The ROC curve, a cutoff of hs-CRP = 7 mg/L, showed a reasonable specificity (76%) for detecting biopsy-proven fibrosis and steatosis.

**Conclusion:**

hs-CRP was associated with any degree of histologically diagnosed liver damage, and it had a reasonable specificity for predicting biopsy-proven steatosis and fibrosis in obese individuals. Further studies are needed to identify non-invasive biomarkers that could predict NALFD progression due to the relevant health risks linked to liver fibrosis.

## Keypoints

- Obesity and NAFLD/NASH are commonly accompanied by inflammation.
- We tested if serum hs-CRP is associated with indices of NAFLD/NASH and liver fibrosis in morbidly obese patients.
- hs-CRP was significantly associated with any degree of histologically diagnosed liver damage.

## Introduction

Non-alcoholic fatty liver disease (NAFLD) is the commonest cause of chronic liver disease worldwide. It is a growing public health problem due to its rising prevalence and associated risk of cirrhosis and cardiometabolic diseases [1–3]. Indeed, NAFLD can progress to steatohepatitis (NASH), and hepatocellular carcinoma (HCC) [4]. Therefore, the search for simple, non-invasive, and low-cost biomarkers that could predict NAFLD/NASH is essential, especially considering the absence of liver-sensitive and specific indicators of these diseases.

Inflammation is present in the early phase of NAFLD and is an essential driver in the initiation and progression of liver damage [4]. A series of proinflammatory proteins and cytokines are implicated in hepatic inflammation have been studied to test their usefulness as non-invasive soluble biomarkers for NAFLD/NASH diagnosis and prognosis [5–23]. Direct associations with various inflammatory cytokines [6, 18, 20, 21, 23], have been reported. High-sensitivity C-reactive protein (hs-CRP) is one of the essential acute-phase proteins. It is now considered a disease marker in many chronic, non-communicable diseases, such as cardiovascular and dysmetabolic diseases [24, 25]. Unlike the inflammatory markers mentioned above, CRP is a low-cost biomarker assayed in most laboratories. Most previous studies [5, 9–11, 13–19] have found a predictive role for CRP in diagnosing NAFLD/NASH. Several studies have reported that hs-CRP can be utilised as a marker of NAFLD/NASH severity/progression [7, 12], while other investigations have failed to find such an association [9, 14, 21, 22]. Unfortunately, most of these studies did not use liver biopsy, the gold standard test for diagnosis; instead, they used less-specific diagnostic criteria, such as elevated levels of alanine aminotransferase and sonographic findings [5, 8, 10–13, 15, 17–19]. Therefore, additional research is required to elucidate the specificity and sensitivity of hs-CRP as a non-invasive marker for the presence, severity, and progression of NAFLD/NASH.

This observational study aimed to assess the concordance between hs-CRP concentrations and liver steatosis and fibrosis/NASH indices based on liver biopsy, elastography, and sonography findings in individuals who were bariatric surgery candidates.

## Patients and methods

This study included individuals with severe obesity [body mass index (BMI) over 40 kg/m^2^ or over 35 kg/m^2^ with comorbidities who were referred to our clinic. Informed written consent was also obtained from each participant. Inclusion criteria were males and females with alcohol consumption less than 30 g/day and 20 g/day, respectively, no (or temporary) use of hepatotoxic drugs, and no hepatitis B or C viruses as confirmed by specific antibodies. Ultimately, 90 patients were selected for the study.

### Serum markers

Fasting blood samples were obtained from participants. The initial laboratory tests were as follows: hs-CRP, fasting blood glucose, insulin levels, aspartate aminotransferase, alanine aminotransferase, gamma-glutamyltransferase and alkaline phosphatase.

#### Two-dimensional shear wave elastography

Two weeks before liver biopsy, two-dimensional shear-wave elastography was performed to determine liver stiffness. This novel technique used an Aixplorer ultrasound system and a convex broadband probe (SC6-1, 1–6 MHz) and was performed according to the instructions provided by the manufacturer. A total of 10 image acquisitions from each subject was considered a good liver stiffness measurement (LSM). In addition, the mean value (M), in kilopascals (kPa), of the liver stiffness evaluation (LSE) was reported for each individual. The technicians performing such evaluations were blinded.

#### Histologic analysis of the liver

A liver biopsy was taken from the left hepatic lobe during bariatric surgery. The indications for biopsy include elevated liver function tests, hepatic steatosis/dysmorphism confirmed via ultrasound or gross damaged liver tissue discovered during surgery, who was blinded to the elastography measures. Paraffin-embedded specimens were stained with Masson’s trichrome, hematoxylin–eosin-saffron, and picrosirius red. The pathologist who assessed the biopsies was also blinded to all data. The NASH Clinical Research Network Modified Brunt approach and the NASH Activity Score (NAS) were used to evaluate NASH [26].

**Fig. 1:**
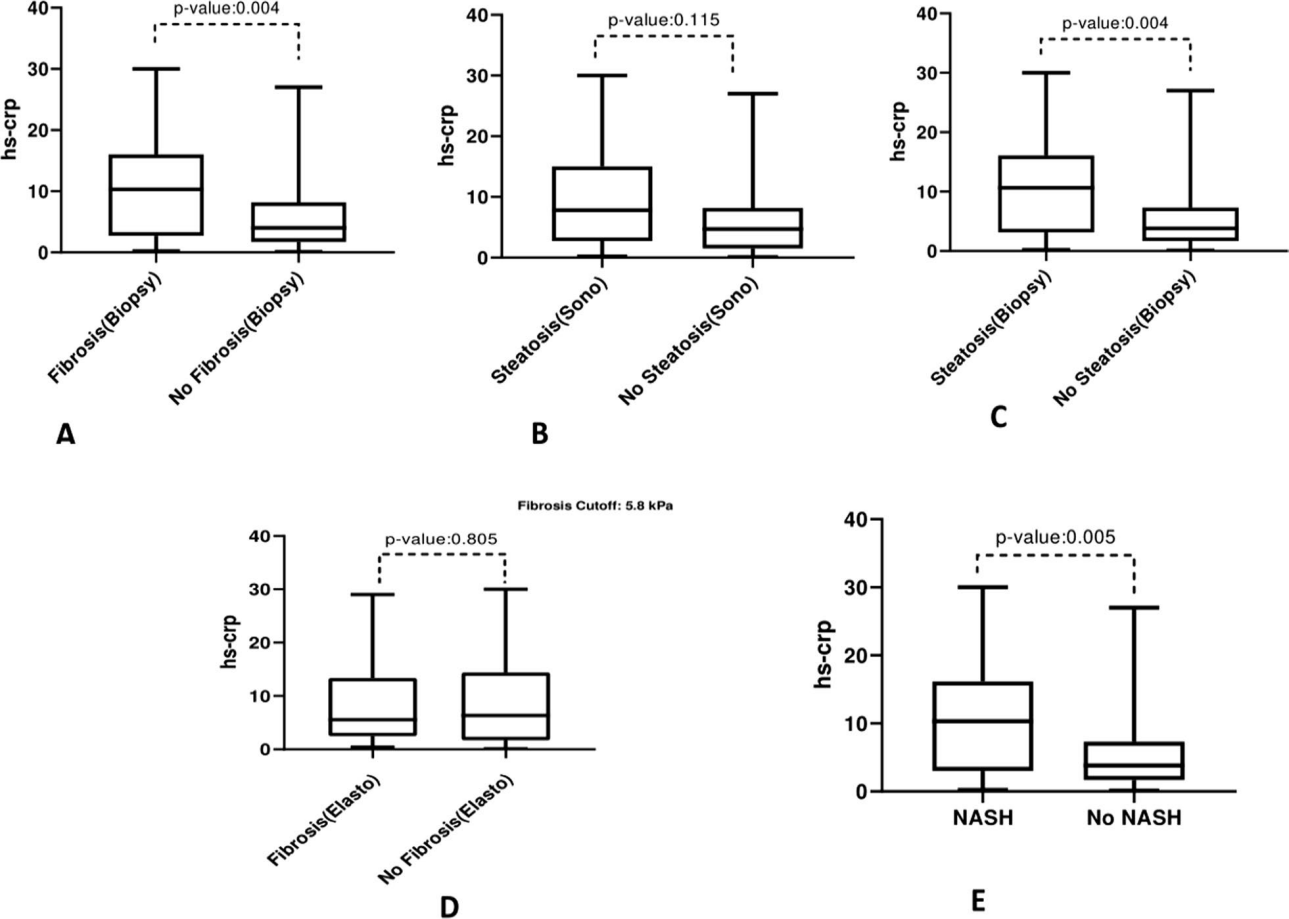
Median (IQR) of serum hs-CRP in liver disease: liver fibrosis at histology (**A**), liver steatosis at ultrasonography (**B**), liver steatosis at histology (**C**), liver fibrosis at elastography (**D**), and NASH (**E**). IQR: Interquartile range; hs-CRP: high-sensitivity C-reactive protein; NASH: non-alcoholic steatohepatitis

Each hepatic disorder was denoted by a scoring system based on 2D-SWE results as follows: five stages in hepatic fibrosis (scored from 0 to 4), percentage of involved portions of the liver in hepatic steatosis [scored from 0 to 3 (0, < 5%; 1, 5–33%; 2, 34–66%; 3, > 66%)], number of diagnosed foci in a ×20 magnification of lobular inflammation [scored from 0 to 3] and number of ballooned hepatocytes with hepatocellular ballooning [scored from 0 to 2]. The NAS score for each patient was calculated by adding all of the scores mentioned above. Based on the total points calculated, patients were then classified into three groups as follows; no NASH (0–2 points) and definite NASH (3–8 points) [26, 27]. Our proposed optimal cutoff value of 2D-SWE for the detection of *F* > 0 was 5.8 Kpa (Fig. 1).

### Statistical analysis

Spearman's coefficient was used to assess and display the relationship between ordinal data. To examine differences in nonparametric data, the Kruskal–Wallis statistical test was applied. DeLong's approach for correlated data was also used to calculate sensitivity, specificity, and areas under the ROC curves for the corresponding data. A binary logistic regression model adjusted for confounders was used to predict the relationship between hs-CRP as a predictor and the continuum of fatty liver disease.

## Results

### Patient characteristics

Out of 111 participants, 90 met the inclusion criteria. The mean age of patients was 38.5 ± 11.1 years, and the mean BMI was 45.46 ± 6.26 kg/m^2^. 51.9% of participants met the criteria for metabolic syndrome; the patients were categorised based on with (*F* ≥ 1) or without (*F* < 1) fibrosis. There were 38 patients and 52 patients without fibrosis. Severe steatosis (defined as > 66% steatosis) was identified in 8.9%, and NASH was detected in greater than 50% of the participants (Table 1).

**Table 1:** Patient characteristics

Variable	Total
Male	18 (20)
Age (years)	38.5 ± 11.1
BMI (kg/m^2^)	45.46 ± 6.26
Weight (kg)	121.34 ± 20.32
Waist Circumference (cm)	133.04 ± 13.6
Height (m)	1.62 ± 8.87
Diabetes Type 2	25 (27.8)
Hypertension	23 (25.6)
Metabolic syndrome	46 (51.1)
Liver stiffness measurement (kPa)	6.1 ± 1.25
Body fat per cent, %	46.72 ± 5.47
Fat mass, kg	56.62 ± 11.58
Fat-free mass, kg	64.51 ± 12.35
Hs-CRP (mg/L)	5.05 (2.4—13.6)
Fibrosis stage	
0 = No fibrosis	38 (42.2)
1 = Zone 3 perivenular or pericellular fibrosis	40 (44.4)
2 = Stage 1 plus portal fibrosis 3 = Bridging fibrosis, focal or extensive	8 (8.8) 4 (4.4)
4 = Residual pericellular fibrosis	–
NASH	
No NASH (0–2)	39 (43.3)
NASH (3–8)	51 (56.7)
Steatosis	
S0 = < 5%	39 (43.3)
S1 = 5–33%	31 (34.4)
S2 = 34–66%	12 (13.3)
S3 = > 66%	8 (8.9)

### Hs-CRP concentration according to fatty liver disease

The comparison of serum hs-CRP concentrations between study subgroups is presented in Fig. 1 (A–E panels). Liver fibrosis (fibrosis < 1 vs. fibrosis ≥ 1), steatosis (steatosis ≤ 1 vs. steatosis > 1), and NASH (0–2 vs. 3–8) in histology, liver fibrosis at elastography (< 5.8 kPa vs. ≥ 5.8 kPa) and liver steatosis at ultrasonography were categorised in A–E panels, respectively. The median (IQR) serum hs-CRP levels in patients with positive histology for steatosis, liver fibrosis, and NASH were significantly greater than those with negative histology (*p* value = 0.004, *p* value = 0.004, and *p* value = 0.005, respectively). Also, serum hs-CRP concentrations were not significantly different in patients with positive and negative elastography.

### Differences of serum hs-CRP between liver disease stages based on histology

The Kruskal–Wallis test showed a significant difference in the serum hs-CRP between liver steatosis stages (*p* = 0.030). Also, there was a significant difference in hs-CRP among liver fibrosis stages (*p* = 0.023). Moreover, there was a significant difference in hs-CRP among NASH stages (*p* = 0.013).

### hs-CRP and liver disease

The relationship between hs-CRP and NASH, liver fibrosis, liver steatosis measured by histology, liver elastography, and ultrasonography are indicated in Table 2. Hs-CRP levels had positive correlation with NASH and steatosis at histology (*p* value = 0.042, *p* value = 0.011, respectively). There were no correlations between hs-CRP levels and liver fibrosis (histology and elastography) as well as steatosis.

**Table 2:** Correlation coefficient between parameters

Correlation coefficient	hs-CRP	
	*R*	*p* Value
Fibrosis (biopsy)	0.216	0.054
NASH score (biopsy)	0.230	0.042
Steatosis (biopsy)	0.284	0.011
Elastography	0.045	0.690
Ultrasonography	0.191	0.093

*NASH* Non-alcoholic steatohepatitis

### hs-CRP and anthropometric indices

In Table 3, body fat per cent showed a significant positive correlation with serum hs-CRP (*r* = 0.240; *p* = 0.014).

**Table 3:** Correlation between CRP and anthropometric indicies

Correlation coefficient	hs-CRP	
	*R*	*p* Value
Body fat per cent, %	0.240	0.014
Fat-free mass, kg	0.007	0.950
Fat mass, kg	0.213	0.069

### Diagnostic value of hs-CRP

**Fig. 2:**
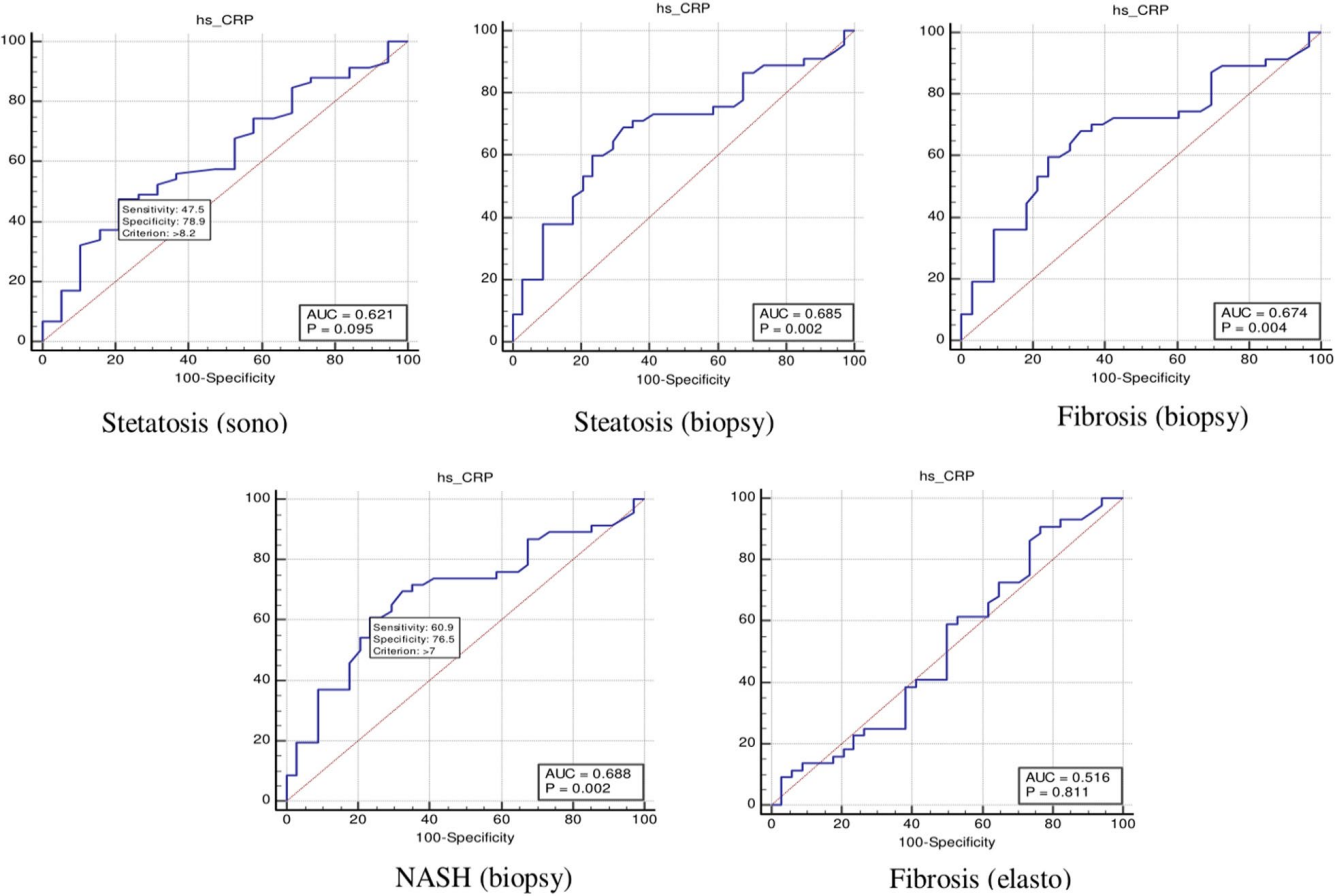
The receiver operating characteristic (ROC) curve for serum hs-CRP in detection of liver fibrosis (histology and elastography), steatosis (histology and elastography) and NASH

In Table 4, the ROC curves at optimal cutoff points determined the diagnostic values. Additionally, both specificity and sensitivity values for each NASH CRN-modified BRUNT methodology stage are summarised. ROC curves were utilised to recognise the sensitivity and specificity of serum hs-CRP for liver fibrosis (histology) (*p* value = 0.004), NASH score (*p* = 0.002), liver steatosis (histology) (*p* = 0.002), liver fibrosis (elastography) (*p* value = 0.011), and liver steatosis (ultrasonography) (*p* = 0.095) (Table 4 and Fig. 2). According to the ROC curves, the optimal cutoff values for levels of hs-CRP necessary for the detection of fibrosis (histology), NASH, steatosis (histology), fibrosis, and steatosis were 7, 4.7, 7, 1.5, and 8.2, respectively.

**Table 4:** hs-CRP in liver disease

Diagnostic performance	AUC	Cutoff	Sens (%)	Spec (%)
Hs-CRP				
Fibrosis (biopsy)	0.67	7	60	76
NASH score (biopsy)	0.68	4.7	69	68
Steatosis (biopsy)	0.68	7	61	76
Fibrosis (Elastography)	0.51	1.5	91	24
Steatosis (ultrasonography)	0.62	8.2	48	79

NASH: Non-alcoholic steatohepatitis; hs-CRP: high-sensitivity C-reactive protein;

### Logistic regression analysis

After adjusting for age, sex, lipid drug, lipid profile, AST, WC, ALT, ALP, GGT and HOMA-IR in the subject groups, binary logistic regression analysis for hs-CRP is shown in Table 5. As shown in regression analysis, in both adjusted and unadjusted models, serum hs-CRP levels were a predictive factor for liver histology (unadjusted model; *p* = 0.009, *p* = 0.006, and *p* = 0.005 for fibrosis, NASH and steatosis, respectively; whereas, in the adjusted model, the *p* values were *p* = 0.024, *p* = 0.014, and *p* = 0.014 for fibrosis, NASH, and steatosis, respectively).

**Table 5:** Binary logistic regression analysis—hs-CRP and study parameters

Parameters		*P*	OR	95% CI for OR	
				Lower	Upper
Crude model	NASH score (biopsy)	0.006	1.10	1.029	1.183
	Fibrosis (biopsy)	0.009	1.09	1.024	1.117
	Steatosis (biopsy)	0.005	1.10	1.030	1.186
	Fibrosis (Elastography)	0.927	1.003	0.950	1.058
	Steatosis (ultrasonography)	0.105	1.066	0.987	1.152
Adjusted model	NASH score (biopsy)	0.010	1.17	1.030	1.360
	Fibrosis (biopsy)	0.014	1.16	1.039	1.327
	Steatosis (biopsy)	0.010	1.174	1.039	1.327
	Fibrosis (Elastography)	0.921	0.004	0.925	1.090
	Steatosis (ultrasonography)	0.326	1.051	0.952	1.161

ALP: ALT: alanine aminotransferase, alkaline phosphatase, hs-CRP: high-sensitivity C-reactive protein, AST: aspartate aminotransferase, Chol: cholesterol; GGT: gamma-glutamyl transferase, HDL: high density lipoprotein; HOMA-IR: homeostatic Model Assessment for Insulin Resistance, LDL: low-density lipoprotein; NASH: non-alcoholic steatohepatitis, TG: triglyceride; WC: waist circumference

## Discussion

The present study showed that hs-CRP values were associated with biopsy-proven liver steatosis, NASH, and fibrosis. A hs-CRP cutoff < 7 mg/L had a reasonable specificity; that is, it allows for correct reporting of approximately 80% of patients without documented liver steatosis or fibrosis at biopsy as true negative.

Adipose tissue dysfunction associated with obesity, adipocyte hypertrophy, and hyperplasia causes a low-grade systemic inflammation characterised by increased pro-inflammatory molecules. Accordingly, many of our patients showed increased hs-CRP levels (78% above 2 mg/L, 70% above 3 mg/L, and 38% above 10 mg/L). Previous studies reported that low-grade inflammation and increased circulating concentrations of proinflammatory cytokines are associated with visceral adipose tissue [28]. Similarly, in our studied population, hs-CRP values and body fat percentage were positively correlated.

NAFLD is the result of hepatic lipid accumulation due to increased free fatty acids (FFA) derived from one or more of the following mechanisms; (1) increased lipolysis in adipose tissue, (2) reduced FFA oxidation, (3) enhanced de novo hepatic lipogenesis, and (4) reduced hepatic very-low-density lipoprotein-triglyceride secretion [4]. Multiple insults (adipokine secretion, inflammation, lipotoxicity, deregulation in glucose and lipid metabolism) triggered by liver lipid accumulation might act synergistically to determine the progression from NAFLD to NASH/cirrhosis [4, 29]. The progression to NASH is associated with systemic inflammation and underpinned by many processes, such as endoplasmic reticulum stress, adipocytokine deregulation, mitochondrial dysfunction, alterations in innate immunity, and toll-like receptor signalling, and intestinal dysbiosis. These processes lead to the accumulation of extracellular matrix, fibrosis development, and liver function deterioration [4, 29]. These pathological conditions (dysbiosis, inflammation, insulin resistance, adipocytokine deregulation) are common in severe obesity. Accordingly, almost 60% of our patients showed some degree of histologically-diagnosed liver damage.

Inflammation can potentially be perpetuated through a vicious cycle that causes further hepatocyte damage. Consistently, we found a significant association between the acute-phase protein hs-CRP and every degree of biopsy-proven liver damage (steatosis, NASH, and fibrosis), in line with the literature [5, 7–9, 11–19]. Intriguingly, these associations remained significant after adjusting for waist circumference and the insulin resistance index (HOMA-IR), suggesting that hs-CRP is a marker of liver damage independent of obesity and insulin resistance. However, a study carried out in individuals with type-2 diabetes did not show an association between liver damage and CRP [22]; indeed, treatments, or the underlying conditions, might have masked the relationship. Other previous studies have identified different inflammatory markers (*i.e.*, VCAM-1, IL-6, IL-8, chemokines) with better performance than hs-CRP in distinguishing advanced fibrosis from milder stages [6, 18, 20, 21, 23]. However, these markers are not assayed in all laboratories, are rarely used in clinical practice, and are very expensive.

This is the first study that simultaneously compared different methods to define the diagnostic accuracy of hs-CRP in the detection of liver disease. In addition to the most widely used technique in clinical practice (ultrasonography) and the ‘gold standard’ (liver biopsy), 2D-SWE was employed. This diagnostic tool was recently shown to have good accuracy in hepatic function and fibrosis assessment in severely obese candidates for bariatric surgery [30, 31].

In our studied population, hs-CRP values showed an acceptable specificity towards liver steatosis or fibrosis. This association may be attributed to inflammatory process and insulin resistance along with dyslipidemia, excess weight, and increased liver enzymes [32, 33].

These results are intriguing considering the difficulty of obtaining morphological liver assessments in patients with severe obesity [34]. However, liver biopsy is a high-cost, invasive technique, afflicted by sampling error, inter-operational variability, and complications because a thick subcutaneous layer of fat and poor mechanical beam transmission make non-invasive imaging methods less accurate in the presence of severe obesity [34, 35]. Furthermore, the measurement of other non-invasive biomarkers (e.g., blood transaminases) provides non-specific indications regarding the type and extent of liver damage. To overcome the limitations of these methods, simple non-invasive fibrosis scores employing readily available laboratory parameters have been developed (*i.e.*, AST/ALT ratio, APRI, FIB-4, NFS, BARD, etc.) [36]. However, their usefulness in predicting liver fibrosis has recently been questioned, especially in severe obesity [36, 37].

In our patients, hs-CRP showed a lower specificity towards NASH prediction, in line with two other studies, showing that this protein could not differentiate steatohepatitis from simple steatosis [9, 14]. Therefore, inflammation was hypothesised to precede liver steatosis, and the common pathogenesis might decrease the discriminatory role of hs-CRP for steatohepatitis [4, 9]. However, CRP mRNA expression in the liver is significantly elevated in NASH patients compared to patients with simple steatosis, thus, suggesting a pathogenetic implication of this protein in steatohepatitis [7].

Finally, around 20% of our patients without the disease were incorrectly identified to test positive. Genetic/epigenetic/environmental factors, and several protective mechanisms, including liver triglycerides, increased adiponectin levels, and hyperleptinemia, might potentially be involved in protecting the liver from toxic lipid insults [4]. The pathogenesis of NAFLD is a complex clinical course involving different processes and pathways that need to be better characterised to develop new non-invasive markers of NASH and fibrosis, which can simultaneously take into account the numerous implicated factors in the pathogenesis of NAFLD.

NAFLD is associated with an increased risk for cirrhosis, cancer, and cardiometabolic diseases [1–3, 13, 38]. Correct and accurate evaluation of the histological stage is crucial since fibrosis represents an adverse prognostic factor. Therefore, it is essential to identify non-invasive biomarkers for predicting the severity of NASH and liver fibrosis. Hs-CRP is a readily-available and straightforward marker but showed low sensitivity in our patients with severe obesity and other cohorts from previous studies [10, 19, 21, 23]. From a clinical perspective, there probably is no single biomarker alone that can differentiate the severity of the liver disease. New insights into the pathogenesis and progression of NAFLD will help search for a combination of predictive non-invasive biomarkers, which must be validated as accurate indicators for both disease prognosis and the response to treatment.

It is worth noting that the availability of histology may be considered a strength of the study because we obtained liver samples during the surgery, which were larger than percutaneous samples (50 > mm vs. > 25 mm). Furthermore, an expert surgeon obtained the liver specimen under gross examination during surgery to take the most optimal specimen. One limitation to our study was that our sample size was small; specifically, only a few patients showed fibrosis at the time of the histological exam/assessment. This may be due to their relatively young age since age is one of the most important variables influencing NASH onset and its development [37]. The observational nature of our study also precludes interpretations of causality.

## Conclusions

Our results suggest that hs-CRP was associated with any degree of biopsy-proven liver damage. Furthermore, it was reasonably specific for predicting biopsy-proven steatosis and fibrosis in severe obesity. There is a need to search for non-invasive biomarkers that can predict NALFD progression due to the relevant health risks linked to liver fibrosis.

## Data Availability

Not applicable.
